# Epicardial Adipose Tissue in Patients with Chronic Obstructive Pulmonary Disease

**DOI:** 10.1371/journal.pone.0065593

**Published:** 2013-06-06

**Authors:** Jorge Zagaceta, Javier J. Zulueta, Gorka Bastarrika, Inmaculada Colina, Ana B. Alcaide, Arantza Campo, Bartolome R. Celli, Juan P. de Torres

**Affiliations:** 1 Pulmonary Department, Clinica Universidad de Navarra, Pamplona, Spain; 2 Radiology Department, Clínica Universidad de Navarra, Pamplona, Spain; 3 Internal Medicine Department, Clinica Universidad de Navarra, Pamplona, Spain; 4 Pulmonary Division, Brigham and Women’s Hospital, Boston, Massachussetts, United States of America; I2MC INSERM UMR U1048, France

## Abstract

**Rationale:**

Epicardial Adipose Tissue (EAT) volume as determined by chest computed tomography (CT) is an independent marker of cardiovascular events in the general population. COPD patients have an increased risk of cardiovascular disease, however nothing is known about the EAT volume in this population.

**Objectives:**

To assess EAT volume in COPD and explore its association with clinical and physiological variables of disease severity.

**Methods:**

We measured EAT using low-dose CT in 171 stable COPD patients and 70 controls matched by age, smoking history and BMI. We determined blood pressure, cholesterol, glucose and HbA1c levels, microalbuminuria, lung function, BODE index, co-morbidity index and coronary artery calcium score (CAC). EAT volume were compared between groups. Uni and multivariate analyses explored the relationship between EAT volume and the COPD related variables.

**Results:**

COPD patients had a higher EAT volume [143.7 (P_25–75_, 108.3–196.6) vs 129.1 (P_25–75_, 91.3–170.8) cm^3^, p = 0.02)] and the EAT volume was significantly associated with CAC (r = 0.38, p<0.001) and CRP (r = 0.32, p<0.001) but not with microalbuminuria (r = 0.12, p = 0.13). In COPD patients, EAT volume was associated with: age, pack-years, BMI, gender, FEV_1_%, 6 MWD, MMRC and HTN. Multivariate analysis showed that only pack-years (B = 0.6, 95% CI: 0.5–1.3), BMI (B = 7.8, 95% CI: 5.7–9.9) and 6 MWD (B = −0.2, 95% CI: −0.3–−0.1), predicted EAT volume.

**Conclusions:**

EAT volume is increased in COPD patients and is independently associated with smoking history, BMI and exercise capacity, all modifiable risk factors of future cardiovascular events. EAT volume could be a non-invasive marker of COPD patients at high risk for future cardiovascular events.

## Introduction

Chronic obstructive pulmonary disease (COPD) and cardiovascular diseases (CVD) are two of the top causes of death worldwide [1]. COPD has been described as an independent risk factor for CVD (2) and the latter is a major cause of mortality in COPD, particularly in patients with mild to moderate disease [3]–[5].

Non-invasive CVD markers may be important to identify COPD patients who are at high risk to develop future cardiovascular events. Beyond the traditional non-invasive CVD risk factors, several other have been proposed for COPD patients, including C-Reactive Protein (CRP) [6], arterial stiffness evaluated by pulse wave velocity (PWV) [7], carotid intima-media thickness (IMT) [8], ankle-brachial index (ABI) [9], and microalbuminuria (MAB) [10].

Epicardial Adipose Tissue (EAT) is the visceral thoracic fat located between the myocardium and the visceral pericardium, and given its anatomical proximity to the heart, EAT can locally modulate the myocardium and coronary arteries [11]. Like other white adipose tissue loci, EAT could function as a lipid-storing depot, as an endocrine organ that secretes hormones, and as inflammatory tissue that secretes cytokines and chemokines [12]. EAT volume can be quantified by different non-invasive radiological techniques, such as echocardiography [13], magnetic resonance imaging (MRI) [14], and computed tomography (CT) [15]. Among available imaging modalities, volumetric quantification of EAT with multidetector computed tomography (CT) has been shown to be one of the most reliable and reproducible methods to assess the amount of EAT [16]. Furthermore, the large population based Multi-Ethnic Study of Atherosclerosis (MESA) Study recently confirmed that EAT volume measured with a chest CT, predicted incident coronary heart disease independent of conventional risk factors [17].

We hypothesized that patients with COPD would have larger EAT volumes than appropriately matched controls, and that the EAT volume in the COPD patients would relate to factors known to increase risk for cardiovascular events. To test this hypothesis we designed this cross sectional study that compared current and former smokers with and without COPD, matched for age, smoking history and body mass index (BMI).

## Methods

### Study Population

**Figure 1:**
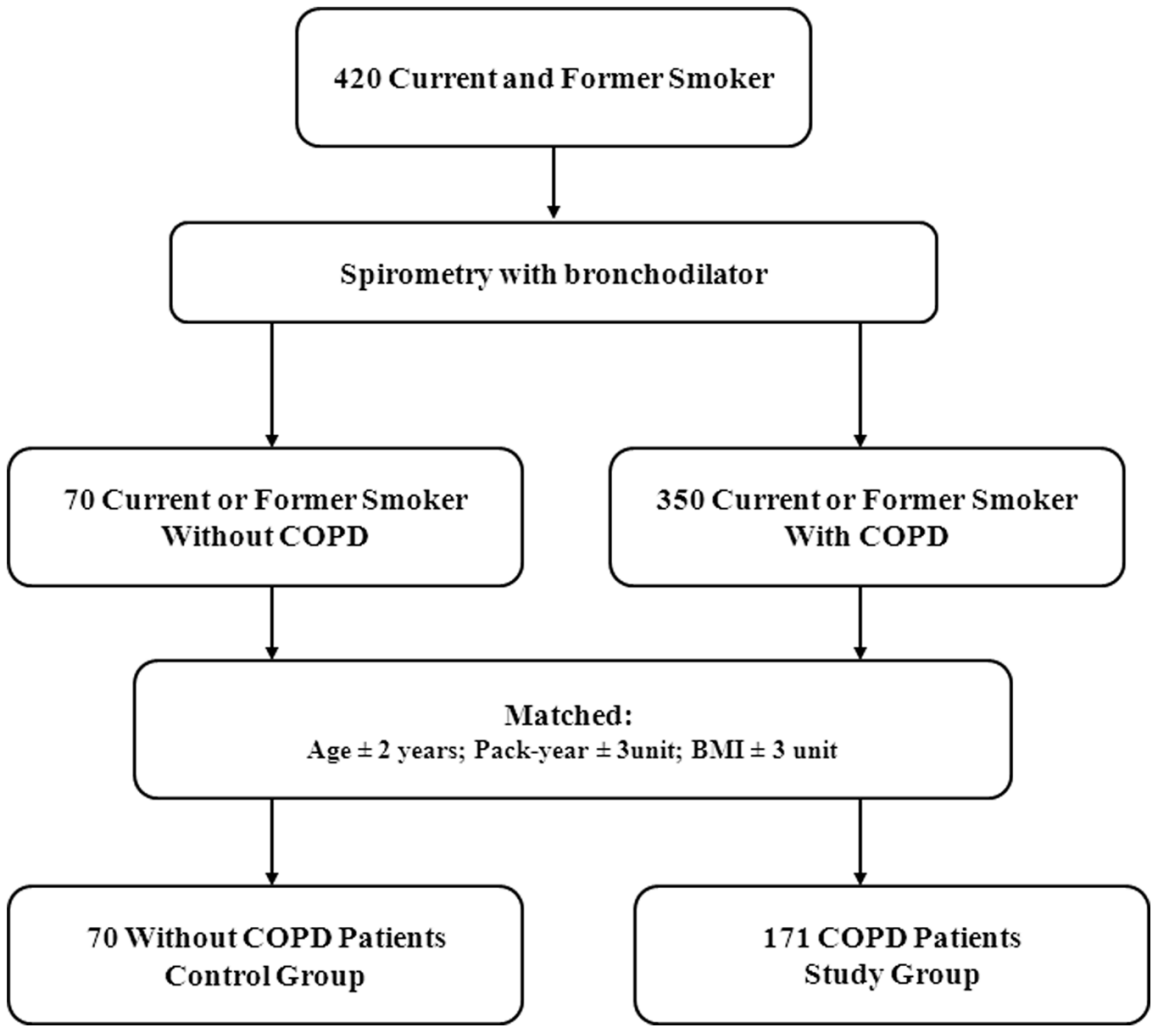
Flow diagram of the study population.

The institutional review board of the Clinica Universidad de Navarra approved this study. Participants were former and current smokers with and without COPD regularly seen in our pulmonary clinic (Figure 1). They all signed the consent form approved by the Human Review Board (Pamplona: “Comité de Etica de la Investigación, Universidad de Navarra IRB n°: 043/2006”). Subjects were consecutively enrolled from January 2002 to August 2012. COPD was defined by a history of smoking more than 10 pack-years and a post-bronchodilator FEV_1_/FVC less than 0.7. To be enrolled, COPD patients had to be clinically stable for 8 weeks prior to entry, and receiving optimal therapy according to international guidelines [18]. The non-COPD group was comprised of smokers and former smokers with a smoking history greater than 10 pack-years and without postbronchodilator airflow obstruction (FEV_1_/FVC>0.7). All postbronchodilation measurements were performed 15 minutes after the inhalation of 400 ug of albuterol. Exclusion criteria were uncontrolled co-morbidities such as malignancy or other confounding diseases. We recorded history of diabetes mellitus, hypertension, dyslipidemia, and the use of anti-hypertensive medications or statins. Blood pressure was measured following standard recommendations [19].

### Matching Process

Figure 1 shows the flow diagram of the matching process. From the initial sample of 420 former and current smokers we identified 70 subjects without COPD and 350 with COPD. For the purpose of this study we assigned former and current smokers without COPD to the control group, and matched each control patient with at least 2 COPD patients with similar age (±2 years), pack-years (±3 pack-years), and BMI (±3 units). Using the aforementioned criteria it was possible to properly match 70 controls with 171 COPD patients.

### Clinical Variables

Lung volumes and spirometry were measured according to ATS/ERS guidelines [20].

The 6-min walking distance (6 MWD) was selected from the better of two walks separated by at least 30 minutes (21). Dyspnea was evaluated by the modified Medical Research Council (MMRC) scale [22]. BMI was calculated in kg/m^2^. The FEV_1_%, BMI, 6 MWD and MMRC values were integrated into the BODE index [23]. The Framingham Score was calculated as previously described [24].

### Laboratory Methods

Morning fasting blood and spot-urine samples were collected simultaneously while at rest and before any other test. The microalbuminuria (MAB) or urinary albumin excretion was determined as the urinary albumin (milligrams) to creatinine (grams) ratio in the morning urine. Urine albumin concentration was determined by a standard turbidometric method (coefficient of variation 5.5%). Serum and urine creatinine concentrations were analyzed using the Jaffe reaction and quantified by a photometric method. Fasting serum levels of glucose, high sensitivity CRP, glycated haemoglobin (HbA1c) and cholesterol were also determined.

### CT Image Acquisition and Reconstruction Protocol

All individuals underwent the CT examinations using a multidetector CT (Somatom Definition and Somatom Sensation 64, Siemens Healthcare, Forchheim, Germany). Low-dose chest CT was performed with 120 kV, 40 mAs, 32×0.6 mm detector collimation, 64×0.6 mm slice acquisition, 0.5 s gantry rotation time, and 1.4 pitch. Images were reconstructed with 5-mm slice thickness using soft-tissue convolution kernel (B31f).

### Epicardial Adipose Tissue Quantification

**Figure 2:**
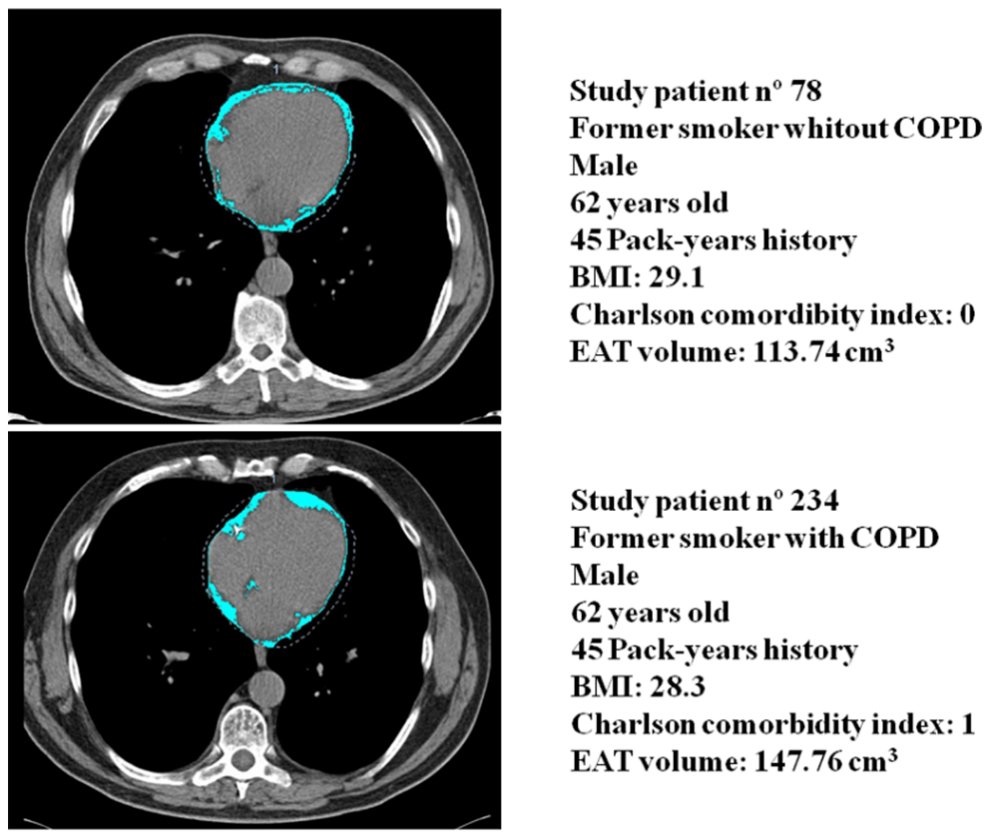
CT of two matched patients participating in the study showing their EAT volume measurements in clear blue.

The amount of EAT volume was assessed by two independent observers (JZ, GB) unaware of the clinical information, using a commercially available software tool (Volume, Siemens) based on attenuation-dependent segmentation methods (Fig. 2). Concordance coefficient between observers was 0.95; with 95% CI between 0.93–0.96. Observers manually traced the pericardium at its superior extent (the center of the right pulmonary artery as it crosses the mid-sagittal plane), at mid-ventricular level, and at the end of the pericardial sac, which defined the inferior extent of the pericardial volume. The software automatically interpolated between the user-defined traces. Automatically traced contours were manually adjusted to the pericardium if needed. Epicardial adipose tissue volume was defined as any fat tissue located within the pericardial sac. A predefined threshold of −195 to −45 HU was used to identify voxels corresponding to fat [17]. Figure 2 shows the CT of two matched patients participating in the study showing their EAT volume measurements.

### Coronary Calcium Calcification Evaluation

Each of the four main coronary arteries was identified (left main, left anterior descending, circumflex, and right). Calcification in each artery was categorized as absent, mild, moderate, or severe and scored by the radiologist as 0, 1, 2, or 3, respectively. Calcification was classified as mild when less than one-third of the length of the entire artery showed calcification, moderate when one-third to two-thirds of the artery showed calcification, and severe when more than two-thirds of the artery showed calcification. With four arteries thus scored, each subject received a CAC score ranging from 0 to 12 [25].

### Statistical Analysis

Data are summarised as relative frequencies for categorical variables, mean (SD) for normally distributed variables and median (25th−75th percentile) for non-normal data. Comparisons between groups were performed using student t-test, Pearson Chi-square or U-Mann Whitney according to the variables type and distribution. A multivariate analysis including Charlson comorbidity index and COPD diagnosis explored the independent association with EAT in the entire population. Other variables with statistical significant difference between groups (lung function, 6 MWD, MMRC, CRP) were not included in the model because of their colinearity with COPD diagnosis.

The associations of the other CVD risk parameters with EAT volume were estimated using Spearman correlation coefficients because of the non-normal distribution of EAT volume values. Linear regression analyses explored the association of each of the studied variables and EAT volume. A multiple linear regression model with EAT volume as the dependent variable and those parameters that showed statistical significance at the level of 0.05 in the univariable analysis was performed to estimate the independent association. Significance level was established as a two-tailed p-Value ≤0.05. Calculations were made with SPSS 15.0, Chicago, U.S.A.

## Results

Seventy current and former smokers without COPD and 171 with COPD participated in the study and their characteristics are shown in Table 1. Although there were patients in every GOLD stage of the 2009 severity classification (I: 44%, II: 36%, III: 16% and IV: 4%), most of them were in GOLD stages I and II. Compared with controls and as expected for a previously matched population, patients and controls had similar age, gender distribution, pack-years history, smoking status, history of diabetes mellitus, cholesterol levels, history of hypertension, blood pressure values and Framingham score. As expected, patients with COPD had abnormal lung function, higher MMRC scores and lower 6 MWD. As previously described [6], [10], other CVD risk markers such as CRP levels and MAB were higher in COPD. Surprisingly, although higher in COPD patients, CAC was not significantly different between the groups. Table S1 shows the characteristics of the COPD patients not selected in the matching process. In comparison to those selected, these patients had the following characteristics: greater age, more pack-years, were less likely to be actively smoking, higher comorbidity index, a similar degree of severity in terms of lung function, less exercise capacity and a higher EAT volume. A multivariate analysis exploring the association of EAT volume with COPD diagnosis after adjusting for Charlson comorbidity showed that the presence of COPD was a statistical significant predictor of EAT volume (β coefficient 28.86 95% CI: 7.6–50.1, p = 0.008).

**Table 1:** Patients characteristics.

Patients characteristics			
	Smoker	COPD	P
n	70	171	
Age (X ± SD)	57±8	59±7	0.056
Gender (%) male/female	80/20	80.5/19.5	1
Pack-year (X ± SD)	39±16	42±17	0.31
Current Smoker (%) yes/no	58.6/41.4	55/45	0,67
Framingham Score (%) Median(p25–p75)	17.3 (10.4–20.8)	17.8 (10.4–28.8)	0.65
Charlson Median(p25–p75)	0 (0–1)	1 (1–2)	<0.001
BMI (X ± SD)	28.2±5.4	26.9±4.8	0.074
FEV_1_/FVC (X ± SD)	75.5±4.8	56.8±10.5	<0.001
FEV_1_ liters (X ± SD)	3.05±0.8	2.18±0.8	<0.001
FEV1% (X ± SD)	99±15.6	73.6±21.9	<0.001
FVC % (X ± SD)	106±17	102.2±20.4	0.17
TLC % (X ± SD)	94.7±13	107.7±14.3	<0.001
MMRC 0–4 (%)	80/18.6/1.4/0/0	44.4/31/15.8/8.2/0.6	<0,001
6 MWD (X ± SD)	539.67±61.7	495.85±100	0.003
BODE Quart 1–4 (%)		82.1/10.3/4.8/2.8	
Hypertension (%) yes/no	45.7/54.2	32.7/67.3	0.08
Anti-hypertensive treatment (%) yes/no	52.9/47.1	65.5/34.5	0.08
SBP mmHg (X ± SD)	127.1±19.4	126.7±19.1	0.86
DBP mmHg (X ± SD)	76.5±12.9	75.22±11.2	0.44
DM (%) yes/no	18.6/81.4	14/86	0.43
Glucose Median(p25–p75)	97 (89.5–108)	98 (90.8–111)	0.36
HbA1c Median(p25–p75)	6 (5.7–6.9)	6.1 (5.6–6.7)	0.84
Dyslipemia (%) yes/no	67/33	71/29	0.35
Anti-hyperlipemia treatment (%)yes/no	63/37	70/30	0.36
Total Cholesterol Median(p25–p75)	202 (185–227)	201.5 (170–231.8)	0.56
LDL-C Median(p25–p75)	123 (104–149)	122 (94.8–157.5)	0.79
HDL-C Median(p25–p75)	56 (45–66)	51 (40–64)	0.12
Albumin/Creatinuria index Median(p25–p75)	6.75 (4.4–12.6)	11.8 (5.1–33.9)	0.008
**EAT cm^3^ Median (p25–p75)**	**129.1 (91.3–170.8)**	**143.7 (108.3–196.6)**	**0.028**
Coronary Calcium Score Median (p25–p75)	1 (0–2.25)	2 (0–3)	0.062
CRP Median(p25–p75)	0.2 (0.1–0.4)	0.3 (0.2–0.7)	0.041
Systemic corticosteroid treatm (%) yes/no	1.4/98.6	4.1/95.9	0.44

n = Number of participants for each group; BMI = Body Mass Index; FEV_1_ =  Forced Expiratory Volume in the fisrt second; FVC = Forced Vital Capacity; TLC = Total Lung Capacity; MMRC = Modified Medical rtesearch Council; 6 MWD  = 6 Minutes Walk Distance; BODE index: BMI, Obstruction, Dyspnea, Exercise; SBP = Systolic Blood Presure; DBP = Dyastolic Blood Presure; DM = Diabetes Mellitus; LDL-C = Low Density Protein; HDL-C = High Density Protein; EAT = Epicardial Adipose Tissue CRP =  C reactive Protein.

X ± SD = means ± Standart Desviation; y/n = Yes/No; p25–p75 =  interquartile range.

The association of EAT volume with other CVD markers in COPD patients was: age (r = 0.26, p<0.001), smoking history (r = 0.27, p<0.001), BMI (r = 0.66, <0.001), arterial hypertension (r = 0.28, p = 0.001), diabetes mellitus (r = 0.19, p = 0.003), CRP (r = 0.32, <0.001), CAC (r = 0.38, <0.001) and MAB (r = 0.12, p = 0.14).

Table 2 shows the univariate analysis exploring the association of each of the studied parameters with EAT volume in COPD and in smokers without COPD. Statistical significance was found for age, pack-years, gender, BMI, FEV_1_%, 6 MWD, MMRC, hypertension, and use of anti-hypertensive medication. In smokers without COPD, we found statistically significant differences in pack-years, gender, BMI, 6 MWD, HDLc and glucose. Table 3 shows the multivariate analysis that determined the best predictors of EAT volume in COPD patients and in smokers without COPD, including those that showed statistical significance in the univariate analysis. Pack-years, BMI and 6 MWD were the best predictors of EAT volume in COPD, and only BMI in smokers without COPD.

**Table 2:** Univariate analysis exploring the independent association of the studied variables with EAT volume in patients with COPD and in smokers.

Univariate with EAT volume as the dependent variable in COPD			
Variable	Coefficient	CI	p
Age	2.7	2.5 to 2.9	<0.001
Pack-years	1.3	0.7 to 2	<0.001
BMI	9.6	7.5 to 11.6	<0.001
Gender (female vs male)	−54.9	−84 to −25.8	<0.001
FEV1%	−0.7	−1.2 to −0.1	0.01
6 MWD	−0.3	−0.4 to −0.1	<0.001
MMRC	17.3	5.4 to 29.1	0.004
HTN (yes vs no)	37.9	13.1 to 62.7	0.003
Charlson	2.2	−4.8 to 9.3	0.54
BODE	5.9	−1.4 to 13.3	0.11
Anti-Hypertensive treatment (yes vs no)	44	19.8 to 68.2	<0.001
Anti-Hyperlipemia treatment (yes vs no)	14.7	−11.1 to 40.6	0.26
Total Cholesterol	−0.1	−0.4 to 0.2	0.58
LDL-C	−0.1	−0.4 to 0.2	0.46
HDL-C	−0,6	−1.4 to 0.1	0.08
DM (yes vs no)	29.8	−4.3 to 64.0	0.08
Glucose	0.3	−0.1 to 0.7	0.14
HbA1c	2.6	−9.1 to 14.5	0.65
**Univariate with EAT volume as the dependent variable in Smokers** without COPD			
**Age**	1.2	−0.3 to 2.6	0.12
Pack-years	1.2	0.4 to 1.9	0.04
BMI	7.3	5.2 to 9.4	<0.001
Gender (female vs male)	−46.9	−78 to –15.8	0.04
FEV1%	−0.4	−1.3 to 0.4	0.3
6 MWD	−0.4	−0.7 to −0.05	0.03
MMRC	24.1	−3.2 to 51.3	0.08
HTN (yes vs no)	25	−1.5 to 51.5	0.06
Charlson	16	−2.5 to 35.5	0.08
Anti-Hypertensive treatment (yes vs no)	22	−4.7 to 48.7	0.11
Anti-Hyperlipemia treatment (yes vs no)	22.9	−5 to 50.7	0.11
Total Cholesterol	−0.2	−0.5 to 0.2	0.3
LDL-C	−0.3	−0.7 to 0.1	0.21
HDL-C	−1.4	−2.3 to –0.6	0.002
DM (yes vs no)	34.8	−1.6 to 71.2	0.06
Glucose	0.7	0.1 to 1.2	0.025
HbA1c	2.8	−17.7 to 23.4	0.78

BMI = Body Mass Index; FEV1% = Forced Expiratory Volume in the first second percent; 6 MWD = Six Minutes Walk distance; MMRC = Modified Medical Research Council Dyspnea Scale; BODE = Body Mass Index, Obstruction, Dyspnea, Exercise; HTN = Hypertension; LDL-C = Low Density Protein Cholesterol; HDL-C = High Density Protein Cholesterol; DM = Diabetes Mellitus; HbA1c = glycosylated haemoglobin.

**Table 3:** Multivariate analysis exploring the independent association of the studied variables with EAT volume in patients with COPD and in smokers.

Multivariate analysis with EAT as the dependent variable in COPD			
Variable	Coefficient	CI	p
Age	1	−0.41 to 2.1	0.06
Pack-years	0.6	0.04 to 1.3	0.035
Gender (female vs male)	−21.4	−44.1 to 1.2	0.06
BMI	7.8	5.7 to 9.9	<0.001
6 MWD	−0.2	−0.3 to −0.1	<0.001
(*) Variables included in the model: Age, Pack-years, Gender, BMI, FEV1%, 6 MWD, MMRC, HTN			
**Multivariate analysis with EAT as the dependent variable in Smokers without COPD**			
BMI	9.6	6.7 to 12.5	<0.001

(*) Variables included in the model: Pack-years, Gender, BMI, 6 MWD, HDL-C, Glucose.

## Discussion

This study showed that in comparison with appropriately matched controls without COPD, Epicardial Adipose Tissue volume is greater in COPD patients. We also showed in these patients, that EAT volume is independently associated with modifiable CVD risk factors like smoking history, BMI and decreased exercise capacity.

Abnormal Visceral Abdominal Tissue (VAT) deposition is receiving increasing attention in patients with COPD for its potential pathogenic role in CVD in these high risk patients [26]. Previous studies have reported increased visceral fat deposition in non-obese mild to moderate COPD patients evaluated with CT [27], [28]. Epicardial Adipose Tissue is the visceral white deposit located between the myocardium and the visceral pericardium that has metabolically active properties due to its anatomical and functional contiguity with the myocardium [12], [13]. Biochemical and thermogenic cardio-protective properties under physiological conditions have been associated with EAT. However, in pathological circumstances, EAT can affect the myocardium and the coronary arteries through vasocrine or paracrine secretion of pro-inflammatory cytokines [29]. EAT has been associated with BMI, VAT, metabolic risk factors, insulin resistance, and coronary artery disease [13], [30], [31], [32]. Recently, it has been shown that EAT volume can independently predict a higher risk of future incident coronary heart disease in community-based adults without a history of CVD [17]. Therefore EAT volume measured on a chest CT has been postulated as a non-invasive tool to identity high-risk CVD patients in whom specific therapies could be implemented.

In the present study we have shown that former and current smokers with a spirometric diagnosis of COPD have increased EAT volumes in comparison to a similar population matched for age, pack-years, BMI, smoking status, and other important CVD risk factors (diabetes mellitus, arterial hypertension, Cholesterol and CRP). Interestingly, COPD patients in the present study have higher median and 25–75^th^ percentile EAT values (143.7; 108.3–196.6 cm^3^) than those reported in large scale population-based studies like the Framingham Heart Study Offspring (mean 124±SD 50 cm^3^) [33], or the MESA study for those with incident coronary heart disease (mean 100±SD 51 cm^3^) [17]. Although there are differences between these trials in their populations (population based *vs.* current/former smokers with and without COPD) and in CT methodology (non-ECG-gated in the Framingham and in our study *vs*. ECG-gated in the MESA study), the fact that EAT volumes in our control group (median 129.1; 25–75^th^ percentiles: 91.3–170.8 cm^3^) are similar to those from the population based studies, supports the importance and validity of our findings.

CT scans of the chest have been traditionally performed in COPD patients to determine the indication for lung volume reduction procedures [18]. Lately, CT has also been proposed for a better characterization of the phenotypic expression of the disease, and some authors suggest this could be used to tailor personalised treatments [34]. Furthermore, after the publication of the results of the NLST study demonstrating that screening a high-risk population decreases lung cancer specific mortality [35], some authors have postulated that individuals with COPD and emphysema could be good candidates for screening programs in view of their higher risk for lung cancer [36]. As the use of CT for COPD patients increases, evaluation of EAT volumes may allow the identification of patients with a higher risk of incident CVD events, while simultaneously evaluating other important prognostic radiologic factors such as emphysema [37], bronchiectasis [38], presence of suspicious nodules [36], or pulmonary artery enlargement [39].

As has been reported in population-based studies, we show that in COPD patients EAT volume was associated with other CVD risk factors. This is important since it implies that the signal found in population samples is reproducible in high risk cohorts such as COPD patients, where a CT is much more likely to be obtained for clinical reasons.

It has been suggested that the CVD risk associated with EAT is due to two mechanisms. The paracrine secretion of pro-inflammatory cytokines (adiponectin, leptin, TNFalfa, IL-1beta, MCP-1, PAI-1, IL-8, IL-6, resistin, angiotensinogen, VEGF) [29], and the direct release of free fatty acids into the vasa vasorum that are then transported downstream into the arterial wall [13]. Therefore, as occurs in the general population [32], the significant association between EAT volume and CAC scores is expected. MAB levels were also increased in the COPD cohort of this study [40]. Interestingly, MAB was not associated with EAT volume. A potential explanation could be that MAB is a measure of general systemic endothelial dysfunction [41], whereas EAT represents a local phenomenon affecting primarily the coronary arteries.

The present study also shows that EAT volume in COPD patients is significantly associated with important CVD risk factors (age, pack-years, BMI, hypertension), and clinical or physiological descriptors of COPD (age, BMI, FEV_1_%, MMRC, 6 MWD). The multivariate model found that the best predictors of EAT volume were smoking history as expressed in pack-years, the BMI and inversely to the 6 MWD. Interestingly in current or former smokers without COPD, EAT volume was only predicted by BMI as previously reported [16]. Our findings have important implications in the daily management of high-risk COPD patients since all of them are modifiable CVD risk factors. There is evidence in that EAT deposition is dynamic and that it could be modified with different interventions such as weight loss achieved by diet [42] or bariatic surgery [30], and by physical activity [43]. Although not yet studied, it is likely that smoking cessation would have similar consequences. Strategies aiming at decreasing BMI will decrease their EAT volume and probably decrease their CVD risk. Perhaps one of the most interesting findings of this study was the indirect association between EAT volume and 6 MWD. We know that 6 MWD reflects daily exercise activity in COPD patients [44]. It is possible that patients with greater 6 MWD do more exercise and as a result have lower EAT volumes.

Age and gender were the other patients’ characteristics that predict EAT volume. Interestingly, Dagvasumberel et al. [45] recently showed that in 90 patients from Japan with moderate to severe coronary artery disease (CAD), EAT volume was higher in men and strongly associated with coronary atherosclerosis. These authors also found that age was among the predictors of EAT volume in their study patients. Therefore, the present study confirms the consistency of these CVD risk factors as predictors of EAT volume as had previously been shown in a different high-risk population.

The reason why EAT deposition is increased in COPD patients is unclear and was not within the scope of the present work. The report that other chronic inflammatory diseases like rheumatoid arthritis, Crohn`s disease and psoriasis have increase deposition of VAT, suggest a link between inflammation and VAT deposition [26]. The finding that the Fat Mass and Obesity (FTO) gene has been positively associated with BMI in COPD patients, suggests a potential genetic origin since this gene has been associated with differential fat deposition in the visceral o subcutaneos stores [46]. In obese non-COPD patients, increased visceral relative to subcutaneous fat has been associated with hypertryglyceridemia and a decreased rate of glucose utilization [47]. Several reports propose the link between intermittent hypoxemia and visceral fat deposition in animal models [48], [49], suggesting the potential role of hypoxic stress in EAT deposition in patients with COPD, although this has not been demonstrated yet in this population.

There are several limitations to this study. Firstly, it was performed in patients from a pulmonary clinic, and with specific clinical and physiological characteristics limited by the randomisation criteries.Therefore conclusions may not apply to COPD patients in general. Secondly, most of the patients participating in this study had mild to moderate disease. Whether the findings can be extrapolated to patients with more severe disease remains unknown. However, there is plenty of evidence that the highest risk for CVD in COPD patients occurs precisely in those with mild to moderate disease [3], [50]. Thirdly, this is a cross sectional study and therefore the association could be viewed as either a cause or a consequence. Also the sample size of the present work could be viewed as a potential limitation, although this is the first report on EAT in COPD patients. Large longitudinal studies should confirm that EAT volume is an independent risk factor for CVD events in COPD patients.

In conclusion, this is the first study showing that EAT volume is greater in patients with COPD and that it is independently associated with important modifiable CVD risk factors. If confirmed this information could have important implications in the overall management strategy of patients with COPD.

## Supporting Information

Table S1 **Characteristics of the COPD patients not selected in the matching process.**
(DOCX)
