# Supplementary material for: Epicardial Adipose Tissue in Patients with Chronic Obstructive Pulmonary Disease
Source: PLoS One. 2013 Jun 6;8(6):e65593. doi: 10.1371/journal.pone.0065593 (PMC3675061; doi:10.1371/journal.pone.0065593)
Supplement: Table S1 — Characteristics of the COPD patients not selected in the matching process. (DOCX) [file pone.0065593.s001.docx]

Appendix

Table 1. Characteristics of the COPD patients not selected in the matching process

| **Patients characteristics** | |
| --- | --- |
|  | **COPD** |
| n | 179 |
| Age (X ± SD) | 64 ± 9 |
| Gender (%) male/female | 83/17 |
| Pack-year (X ± SD) | 51.3 ± 27 |
| Current Smoker (%) yes/no | 49.6/50.4 |
| Framingham Score (%) Median(p25-p75) | 18.3 (10.8-31.7) |
| Charlson Median(p25-p75) | 2 (1-3) |
| BMI (X ± SD) | 27.2 ± 4.6 |
| FEV_1_/FVC (X ± SD) | 55.27 ± 11.3 |
| FEV_1_ liters (X ± SD) | 1.98 ± 0.8 |
| FEV1 % (X ± SD) | 70.1 ± 21.9 |
| FVC % (X ± SD) | 98.9 ± 20.1 |
| TLC % (X ± SD) | 105.8 ± 14.8 |
| MMRC 0-4 (%) | 37/31.3/20.6/8.7/2.4 |
| 6MWD (X ± SD) | 459.36 ± 116.8 |
| BODE Quart 1-4 (%) | 74.1/15.5/6.2/4.1 |
| Hypertension (%) yes/no | 39.4/60.6 |
| Anti-hypertensive treatment (%) yes/no | 61/39 |
| SBP mmHg (X ± SD) | 128.9 ± 18.7 |
| DBP mmHg (X ± SD) | 75.56 ± 10.6 |
| DM (%) yes/no | 14.1/85.9 |
| Glucose Median(p25-p75) | 98 (90-109) |
| HbA1c Median(p25-p75) | 6 (5.6-6.7) |
| Dyslipemia (%) yes/no | 70.4/29.6 |
| Anti-hyperlipemia treatment (%)yes/no | 68.1/31.9 |
| Total Cholesterol Median(p25-p75) | 199 (170-229) |
| LDL-C Median(p25-p75) | 119.5 (94-153.3) |
| HDL-C Median(p25-p75) | 49 (40.5-61) |
| Albumin/Creatinuria index Median(p25-p75) | 13.5 (6-34.5) |
| **EAT cm³ Median (p25-p75)** | **153.3 (114.2-210.6)** |
| Coronary Calcium Score Median (p25-p75) | 2 (1-4) |
| CRP Median(p25-p75) | 0.4 (0.17-0.8) |
| Systemic corticosteroid treatm (%) yes/no | 5.4/94.6 |
| n = Number of participants for each group; BMI = Body Mass Index; FEV_1_ = Forced Expiratory Volume in the fisrt second; FVC = Forced Vital Capacity; TLC = Total Lung Capacity; MMRC = Modified Medical rtesearch Council; 6MWD = 6 Minutes Walk Distance; BODE index: BMI, Obstruction, Dyspnea, Exercise; SBP = Systolic Blood Presure; DBP = Dyastolic Blood Presure; DM = Diabetes Mellitus; LDL-C = Low Density Protein; HDL-C = High Density Protein; EAT = Epicardial Adipose Tissue CRP= C reactive Protein | |
| X ± SD = means ± Standart Desviation; y/n = Yes/No; p25-p75 = interquartile range | |
